# Hospital Climate and Peer Report Intention on Adverse Medical Events: Role of Attribution and Rewards

**DOI:** 10.3390/ijerph18052725

**Published:** 2021-03-08

**Authors:** Xiaoxiang Li, Shuhan Zhang, Rong Chen, Dongxiao Gu

**Affiliations:** 1 School of Business, Anhui University, Hefei 230601, China; rainy08@sina.com; 2 School of Economics, Anhui University, Hefei 230601, China; zhangsh976729@163.com; 3 School of Economics & Management, Hefei Normal University, Hefei 230061, China; chenrong0723@163.com; 4 School of Management, Hefei University of Technology, Hefei 230009, China

**Keywords:** hospital climate, attribution tendency, peer report intention, reward, health professionals

## Abstract

Adverse medical events (AMEs) often occur in the healthcare workplace, and studies have shown that a positive atmosphere can reduce their incidence by increasing peer report intention. However, few studies have investigated the effect and action mechanism therein. We aimed to extend upon these studies by probing into the relationship between hospital climate and peer report intention, along with the mediating effect of attribution tendency and moderating effects of rewards. For this purpose, a cross-sectional survey was administered in a hospital among health professionals. We collected 503 valid questionnaires from health professionals in China and verified the hypothesis after sorting the questionnaires. The results of empirical analysis show that a positive hospital climate significantly induces individual internal attribution tendency, which in turn exerts a positive effect on peer report intention. Contract reward also helps to increase peer report intention, especially for health professionals with an internal attribution tendency. The findings contribute to the literature regarding AME management in hospitals by providing empirical evidence of the necessity for hospital climate and contract reward, and by providing insights to improve their integrated application.

## 1. Introduction

Medical malpractice often involves adverse medical events (AMEs), which negatively affect patients and are costly to hospitals. Hospitals and health professionals always strive to reduce AMEs [1,2,3]. Unfortunately, according to the World Health Organization report in 2019, in high-income countries, as many as 1 in 10 patients is harmed when receiving hospital care. In low- and middle-income countries, the rate of AMEs was around 8%, of which 83% could have been prevented and 30% led to death [4]. All AMEs, regardless of type, should draw more attention [5,6]. Bottom-up reporting AMEs is more effective than top-down perceiving and monitoring [7]. Moreover, health professionals are usually first to involve and perceive adverse events (AEs) and should act as critical information sources of these events [8]. Therefore, hospitals always encourage staff to report AMEs immediately through incentives, liability exemptions, building AME reporting systems, etc. However, self-report initiative of these events is rare [2], because it may lead to negative impacts on respondent persons, or even other most proximal respondent persons due to the blame trap. Peer report or whistle-blowing from the witness seems like another necessary and possible bottom-up reporting channel.

In the era of self-media, increasing numbers of AEs are being reported through whistle-blowing, which has inspired a variety of studies [9,10,11]. Some studies have investigated relationships between organizational factors and adverse event whistle-blowing [12,13], another focused on the characteristics of events or witnesses, such as justice evaluation on organization, target, and severity of AEs [8]. Few studies have analyzed and tested the effects and action mechanisms of how hospital climate affects whistle-blowing of AMEs. Unlike other contexts, AMEs often lead to much worse consequences and attract more attention because they are directly connected to patient life and health, which prevails over everything. However, relatively little is known about whether persons have more tolerant attitudes toward whistle-blowing of AEs in medical and hospital contexts than in others, where whistle-blowers are more easily considered traitors. As such, it is important to explore whether whistle-blowing is more acceptable in hospitals and hospital climates.

It is necessary to note that individuals’ intention and behavior of report are affected by their causal judgment about AMEs. According to the theory of attribution [14], individuals have various explanations for the events, including situational attribution and dispositional attribution [15]. The former believes that AEs are caused by external factors, and those involved in AEs should not take more responsibility, so peer reports will not bring negative effects to colleagues. However, individuals with dispositional attribution overestimate the impact of internal and underestimate the external and believing that peer reporting of adverse events is detrimental to colleagues. Therefore, we conducted further investigations into the mechanisms of attributional tendency mediating the relationship between hospital climate and whistle-blowing regarding AMEs. Moreover, rewards are often regarded as a common method to encourage whistle-blowing AMEs, so we also verified its true effects.

## 2. Conceptual Background and Research Hypotheses

### 2.1. Hospital Climate and Peer Report Intention

Peer report differs distinctly from whistle-blowing, although they have similar connotations [1,9,16]. For peer reports, reporters and reported persons have equal status or positions in organizations, whereas whistle-blower may also refer to two different parties with superior and subordinate relations [1]. In practice, subordinates are only responsible for their work while superiors may be responsible for the whole department or even the whole organization. Different standpoints lead to different views or attitudes toward AEs and related report behaviors. For example, persons often feel a strong sense of identity with their colleagues, and they tend to blame environmental factors (e.g., unreasonable working arrangements or lacking appropriate training) for AEs that absolutely could not be controlled by their colleagues [17]. It seems that peer report only results in punishments of their colleagues instead of addressing a fundamental solution to the problem. Therefore, peer report is less likely to happen than whistle-blowing supervisors. From another viewpoint, fearing revenge from supervisors may weaken related whistle-blowing intention [7]. This implies that we should not confuse whistle-blowing supervisors with whistle-blowing colleagues, namely peer reporters.

According to Schneider’s definition, climate is the mutual “*perceptions of events, practices, and procedures and the kinds of behaviors that get rewarded, supported, and expected in a setting*” [18]. He also proposed that climates should be considered and studied with a particular strategic focus, such as safety climate and adverse event climate. Organizational climate always exerts a strong impact on individuals’ attitudes and behaviors surrounding AEs, and response measures should certainly include AEs report behaviors [19]. Therefore, we focused on this specific climate in hospitals, adverse medical event climate (AMEC), which describes individual mutual perceptions and attitudes toward AEs in the medical environment.

Scholars often categorize organizational climate into positive or negative [20]. In a positive climate, organizations and their managers realize and acknowledge the existence of AEs, and are committed to and engage in informing and improving AE prevention mechanisms and emergency response systems with high compliance and participation in adverse event prevention and response activities [21]. Their managers focus on problem-solving and providing training or precaution programs [13,22], all of which aim to decrease the likelihood and weaken the influence of AEs. Conversely, in a negative climate, organizations and their managers neglect the existence of potential AEs and threats during normal operation. Once these events occur, everybody is concerned about who should be blamed, punished, or even fired, instead of how to right wrongdoings, lessen adverse impacts, and learn from these events.

Similarly, AMEC can be classified as positive or negative. The positive AMEs appear to be a common problem, and hospitals try to deal with them rationally and scientifically, for example, building special management systems that include AME report sub-systems [23]. Health professionals will implement more constructive measures to mitigate negative events, such as being more problem-oriented in the investigation and consulting with each other in response. In this situation, real-time reports help to minimize loss and avoid recurrence instead of placing blame and dispensing punishments as in negative AMEC. Witnesses report to their supervisors, who take remedial action and save the patients who were diagnosed incorrectly or when a diagnosis was missed. Health professionals are inclined to acknowledge and accept peer report behaviors with less hostility directed toward informants in this climate. Similarly, some scholars noted that positive AMEC promotes non-punitive approaches by supervisors to AME, increases mindfulness and perceived importance of peer reports, and advocates for more open and free-flowing information exchanges, which include peer report behavior [10,24].

Alternatively, AMEs in a negative climate are treated passively, which may worsen the situation [23]. The response of supervisors to AMEs influences the related report behavior intention [9]. Witnesses may feel sympathy when they perceive that someone will be punished for these events, which weakens report intention. The passive manner here means greater losses and threats for the reporting person; informants may experience hostility toward them, but no attention is focused on the AMEs or the patients involved. Worrying about retribution is regarded as the largest obstacle to peer reports [9,25]. The primary value of the peer report is to identify and assign blame to those responsible because AMEs may be triggered by a host of factors, and everyone strives for self-preservation instead of working hard to reduce the incidence of AMEs. Therefore, in both negative and poor AMECs, health professionals refuse to report and tend to aim to satisfy everyone (e.g., neglect or cover up) to avoid retribution and isolation.

Taken together, a positive AMEC means more attention is paid to the patients involved and more effective remedial actions are taken, but punishments and retaliations are fewer, which fosters increased report responsibility felt by witnesses, and vice versa. Therefore, we hypothesize the following:

**Hypothesis** **1.** *Peer report intention is accentuated in positive AMEC and attenuated in negative AMEC.*

### 2.2. Mediating Effect of Individual Attribution Tendency

To address AMEs, witnesses quickly seek the cause and identify the possible responsible persons instinctively, namely by causally attributing the effects to a person’s actions, not only limited to the investigation process. Attribution is a complicated cognitive process and involves information collection, analysis, and other complex processes and activities [26]. However, it is difficult for individuals to immediately determine the real cause because they may lack information and judgment ability [27]. In particular, each person’s concerns drive information seeking and viewpoints selection, which affects the judgment of the cause and the responsible persons. In emergency responses to AMEs, health professionals often judge hastily and some critical information may be ignored, omitted, or unobtainable. All of these lead witnesses to be arbitrary and subjective in the attribution process.

As with most other cognitive and perceptual processes, attribution is inevitably fraught with subjectivity [26,28]. Besides AMEs and hospital conditions, individual factors may play important roles in the attribution processes and results, such as attribution tendency or preference. As stated by Rasmussen, it is often hard to pinpoint the real causes because AME investigations are often driven by preconceived notions about their causes, which are always dominated by an attribution tendency [29,30]. Attribution tendency can be understood multi-dimensionally, and the most important one is external/internal [14,15,31]. Specifically, causes of AMEs may be social, cross-organizational, hospital, departmental, individual, or, more often, a combination. Further, there are two attribution tendencies: externally- and internally-oriented attribution. For example, when administering the wrong medication, prescribing the wrong medication, or other AMEs, witnesses with external attribution tend to regard them as results of circumstances (e.g., unclearly labeled medication supply, high work burden) instead of individual factors (e.g., careless, poor medical skill), and vice versa.

Hospitals with a positive climate proactively build rules and regulations to effectively and efficiently guide the management of AMEs, and everyone necessarily strives for preventing and eliminating AMEs. As stated above, positive AMEC means open and free discussion, constructive training, and an effective AMEs management system. With an honest and open attitude toward AEs, individuals believe everyone has responsibility for avoiding, preventing, and responding to AMEs, and understands their roles clearly [13]. Once AMEs happen, the entire whole hospital is inclined to be problem-oriented, showing concern about how to minimize the impact on patients [5]. The witnesses immediately implement corrective or remedial measures with which they are familiar. Consequently, everyone is less likely to shirk their responsibility for AMEs in this climate and they tend to hold an internal attribution tendency [24]. In this case, the possibility of peer report increases for three reasons: (1) As stated by Victor et al., perceived fairness influences peer report intention [32]. Individuals with internal attribution tendencies realize someone should be responsible for the unnecessary suffering of the patients and should pay for their mistakes. Then, uneasiness and guilt for the person doing the reporting are lessened. (2) Internal attribution means that the real causes of these AMEs are wrongdoings, misconduct, or other unexpected behaviors on an individual level [28]. Reporting to supervisors contributes to solving problems by placing more attention on the issue and securing medical resources. Otherwise, if AMEs originate from environments (e.g., poor hospital management systems or medical conditions), then only pointing out the issues may be less effective in an emergency condition. Righting the wrongdoings of individuals is simpler than that of organizations or environments. (3) Compared with external attribution, witnesses with internal attribution tendency are more likely to engage in pro-social behavior, which includes peer report behaviors [33], because they prefer to alleviate patients’ pain, improve medical service, and promote the medical quality of the AMEs.

Alternatively, under a poor AMEC, health professionals are unwilling and/or unable to discuss AMEs openly and freely, let alone provide constructive suggestions about AMEs. Everyone in this situation likely only has a vague understanding of their responsibilities, or even may not be able to distinguish wrongdoings from correct actions, and is unwilling to admit medical misconduct, etc. They often hold an external attribution tendency, which means the hospitals or the environment, instead of the individuals, should pay for AMEs. Witnesses firmly believe that their innocent colleagues will act as scapegoats and be treated unfairly instead of taking additional measures as required to solve the problems or alleviate the suffering of patients. Consequently, peer report intention is weakened in this AMEC. In summary, a positive AMEC will induce internal attribution, and thereby increase peer report intention, and vice versa. Therefore, we hypothesize the following:

**Hypothesis** **2.** *The attribution tendency of AMEs mediates the relationship between AMEC and peer report intention.*

### 2.3. Moderating Effect of Reward

In terms of patient health and safety, responsible persons will be held accountable and punished, especially those who have submitted late reports, concealed reports, and/or omitted details on AMEs. Besides punishment, rewards are often regarded as another common and even more effective incentive method [34], and some organizations have implemented these methods to promote peer report or whistle-blowing [35]. For example, government agencies often encourage citizens to report illegal activity using cash rewards and are responsible to maintain informants’ privacy and personal details. Rewards can be clearly defined and documented, such as the bonus scheme for report behaviors. Individuals may choose to make the effort and subsequently received the bonus as stipulated in documents or contracts [36]. This category of reward is contracted, whereas others may be extra-contractual. For example, report AMEs may help a person to receive appreciation for their courage and responsibility, and even obtain a promotion, which cannot be defined clearly in hospital work contracts or management systems. Taken together, rewards can be divided into two categories, contractual and extra-contractual [37]. Bonus is a typical means of the former while promotion belongs to the latter.

According to the theory of planned behavior, expected return influences behavioral intention [38,39]. If witnesses perceive that peer reporting results in rewards they will have a more positive attitude toward these behaviors, and report intention subsequently increases. Contractual rewards describe predictable bonuses or other forms of reward provided to informants. In addition, one of the main obstacles to peer report is uncertainty about its correctness and morality [1,40]. Besides predicting what informants will receive, rewards also reflect hospitals’ and their managers’ positive attitudes, which strengthens the recognition of the ethicality of peer report. Extra-contract rewards represent appreciation from hospitals and supervisors and prove the worth of peer report. Furthermore, these rewards may include prestige and promotion, instead of retribution, exclusion, or firing, all of which prevent witnesses from reporting.

We predicted that rewards, regardless of contractual or extra-contractual, would align the informants’ interest with those of their hospitals and supervisors, thereby inducing them to report AMEs. We stress the AMEs reporting responsibility of witnesses and that bonuses or other rewards for peer report behaviors in a hospital should be clearly defined. However, empirical studies proving their practical effects are lacking, so we propose the following:

**Hypothesis** **3.** *Both contractual reward and extra-contractual reward increase peer report intention.*

Peer report negatively affects the responsible or suspected persons related to AMEs, and is often hindered by sympathy, guilt, or other emotions experienced by witnesses toward the perceived at-fault party [25]. As discussed above, health professionals with internal attribution tendencies are likely to believe someone ought to be responsible for AMEs and be punished for their actions. Therefore, identifying them will help patients and prevent the recurrence of similar AMEs. This attribution weakens sympathy, guilt, and other similar emotions experienced by informants, and rewards become more attractive in this situation. Moreover, internal attribution tendency illustrates the pro-social character of individuals [33]; thus, rewards, especially extra-contractual rewards, are more attractive because of individual desire for recognition and approval. Otherwise, individuals with external attribution tendency are unwilling or even ashamed of receiving rewards, as someone will be unfairly held accountable for environmental factors [40,41,42]. The greatest value of peer report lies in receiving bonuses, appreciation, and other personal interests, but not in problem-solving and medical treatment improvement. Therefore, the incentivizing effects of rewards are weakened for those with external attribution tendency.

As with other behaviors, a threshold exists for peer report, whereas rewards will lower the threshold because of its benefit to informants, its implications for hospitals, and their managers’ appreciation. Then, the negative consequences of peer report are attenuated, and the effect of stimulative factors appears, which includes internal attribution tendency. Peer report is considered as an extra-role behavior, and related report intention decreases as a stimulus is removed. It does not matter which attribution tendency is held by the health professionals because the difference therein seems immaterial, as in other situations [33,43]. In other words, the effect of attribution tendency on report intention is strengthened by rewards. Generally, rewards, including contractual and extra-contractual rewards, not only increase peer report intention but also enhance the effect of attribution tendency. The hypothesis below is proposed:

**Hypothesis** **4.** *Rewards have interactive effects with attribution tendency on peer report intention.*

**Figure 1:**
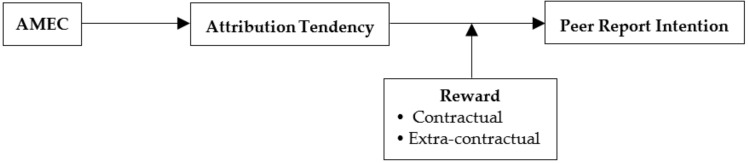
The research model (AMEC = adverse medical event climate).

Based on the above hypotheses, we present our research model in Figure 1.

## 3. Materials and Methods

### 3.1. Study Design

The study was designed as a cross-sectional survey administered to the health professionals of 43 hospitals in the Yangtze River Delta region in China. The primary purpose of the study was to determine whether peer report intention is associated with hospital AMEC. The secondary objective was to analyze the mediating effect of attribution tendency, and the third one was to analyze the moderating effect of contractual and extra-contractual rewards on the relationship between attribution tendency and peer reporting intention.

The advantages of using the hospital context as the study background and health professionals as the study object in studying AEs are threefold: (1) AMEs are closely related to life and health, and hospitals and medical agencies should try everything to effectively avoid, prevent, and respond to them [23]. (2) Medical staff frequently encounter various AEs in their workplaces, and the term adverse event is familiar to them. Nearly all large hospitals have built adverse event report systems, and witnesses are constantly faced with decisions about whether to report. In Britain, for example, the National Centre for Patient Safety (NPSA) was set up in August 2001 to encourage doctors and other staff to report errors and approximations. China has also taken relevant measures. The Hospital Complaint Management Measures norms issued on 26 November 2009, stated that “*health departments at all levels should encourage hospital staffs to report medical errors on their initiative, and gradually establish a non-liability reporting system for medical errors without injury*”. (3) Health professionals and hospitals may also hide and conceal AMEs, which will ultimately worsen the doctor-patient relationship. For example, the results of a survey in the Maternal and Child Care Service Centre of Guangdong Province in China showed that less than 0.5% of AMEs are reported, although these organizations had already implemented AME report systems [44]. Therefore, AMEs provide a unique and suitable research context to analyze and test how factors (e.g., organization climate) influence adverse event report intention.

For the purpose of this study, AME was defined as any inadvertent injury that occurs in hospitals, but AMEs were not explicitly listed because respondents may ignore new or unlisted AMEs. Besides, there are different types and degrees of AMEs in hospitals, and the perception of AMEs of health professionals from different departments may differ. Consequently, a deeper distinction of AMEs was not applied out in this study.

### 3.2. Data Collection

Data collection consisted of two steps. In the first stage, the questionnaires were sent to 3 hospitals (5 persons in each hospital) in Hefei, China for the pre-test. According to the feedback, the items in the questionnaire were repeatedly modified and improved.

In the second stage, questionnaires were distributed to 43 hospitals in the Yangtze River Delta region in Eastern China, including 15 private hospitals and 28 public hospitals. Before visiting each hospital, we took the necessary steps to protect the privacy of the respondents. For example, referring to the specific procedures of Feng and Wu (2005) and other similar studies [45], a sealed questionnaire box placed in convenient locations can increase the willingness to respond. Importantly, these samples were obtained from different departments because the psychological climate of individuals mainly depends on the department climate. Therefore, we contacted 43 doctors (one person in each hospital), each of whom placed 20 boxes in different departments and told colleagues to fill out one questionnaire for each department. For measuring the variable on the organization level, each questionnaire had five copies of the AMEC scale for five health professionals to complete separately, and the average values of the copies represented the AMEC in the workplace. Considering the sensitive nature of peer reports and AMEs, we stressed that the questionnaire was anonymous and only for academic research. As the respondents had to take spare time to fill the questionnaires anonymously, the sealed questionnaire boxes were requested to be returned after two days to ensure responders had enough time to adequately respond to the survey.

**Figure 2:**
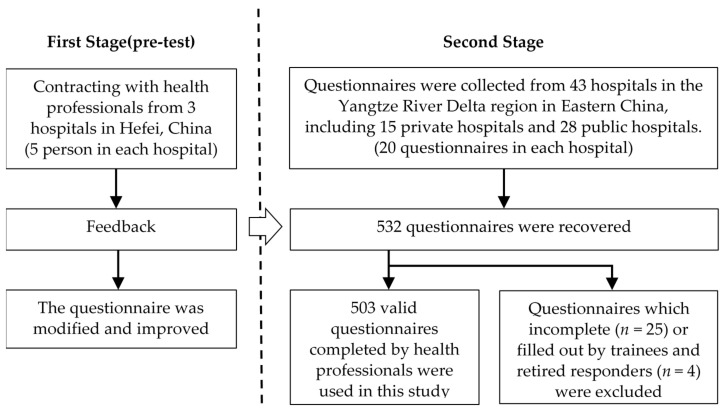
The process of data collection.

In total, 532 health professionals were included in the study (total response rate = 61.9%). Incomplete questionnaires (*n* = 25) or questionnaires filled out by trainees and retired health professionals (*n* = 4) were excluded from this analysis. A total of 503 questionnaires were eligible for this analysis (valid response rate = 58.5%). Figure 2 describes the main data collection process.

### 3.3. Study Participants

A total of about 1850 health professionals participated in this study, of whom 1662 completed validated questionnaires. As there were 503 valid questionnaires and the hospital’s AMEC of one respondent was reflected by the average of three to five colleagues (including this respondent) from the same department, we analyzed the 503 health professionals of the 43 hospitals whose number of respondents per hospital was seven or above.

### 3.4. Scale

#### 3.4.1. Measures

Maturity scales of the relevant variables were used to form the initial questionnaire in this study. Due to the lack of widely used relevant Chinese questionnaires, we reviewed the English literature and asked professionals to translate them to ensure the accuracy of scales. According to the pre-test results, we modified the construction of the questionnaire, as shown in Table A1 of Appendix A.

To measure the degree of peer report intention, respondents are usually required to directly measure report willingness or behavior [1,24], namely, to provide answers to “I will report AMEs that occurred in the hospital if I know”, using a seven-point judgment from strongly disagree to strongly agree.

AMEC was measured using an adaption to Dedobbeleer and BeLand’s nine-item, seven-point scale [46], and modified in detail according to Curran et al. (2018) [47]. Examples of the items are: “how much do your supervisors seem to care about AMEs?”, “how much is management committed to AMEs in your hospital?”, “how important is preventing AMEs to hospital management?”, “do you view AMEs responding as an integrated part of your job?”, and “do you perceive that you will be involved in an AME in the next 12 months?”. The higher value, the more positive AMEC felt by the respondent in the workplace. The Cronbach’s α of this scale was 0.868 and all factor loadings were above 0.7.

A seven-point scale with six “strongly disagree/strongly agree” items were used to assess the internal/external attribution tendency; the higher the score, the great the internal attribution tendency. This scale was modified from Hofmann and Stetzer’s study and mainly referred to judgments and suppositions of AME causes [13]. Examples include “AMEs are usually caused by health professionals’ carelessness and malpractice (reverse-coded)”, “it is hard for health professionals to influence and control whether AMEs occur”. Cronbach’s α of this scale was 0.820, and all factor loadings were above 0.7.

Some hospitals had implemented cash bonus incentive programs to encourage peer reports, but directly obtaining extra-contractual reward information from hospital management documents or systems is difficult. Hence, we asked respondents to rate the likelihood of rewards they would or expect to receive for peer report behaviors. The measurements were directly transferred from the definitions of related concepts. Examples of items were “informants will receive a cash reward for peer report of AMEs”, and “peer report of AMEs will create a favorable impression of informants in the supervisors, which may help with a promotion in the future.”

Some other control factors also possibly influenced peer report intention: (1) Education. Most medical education affects AME management and the response procedures of health professionals, which includes AMEs peer report. Four levels of education were coded using 1 to 4 with 1 indicating junior college or below, 2 representing junior college, 3 representing undergraduate, and 4 denoting a master’s degree or above. (2) Seniority. The longer a person works in the hospital, the more AMEs they experience. Therefore, attitudes toward AMEs and peer reports probably changed according to seniority, which was measured by working years and within the range of 0 to 40. (3) Ownership. Some studies reported that safety management and peer reports are influenced by hospital characteristics [6,16]. Therefore, we selected ownership as one of the control variables, with 0 denoting private hospitals and 1 denoting public hospitals. (4) Scale. The number of hospital beds was used to measure the hospital scale, with 1 indicting 100 or fewer beds, 2 indicating 101 to 500 beds, 3 indicating more than 500 beds. This division of the hospital scale is the most popular in China. (5) Work stability. In public hospitals, some staff is permanent while others are not, so employment forms often represent work stability in these hospitals. However, this distinction does not exist in the employment policy of private hospitals. Therefore, we abandoned this objective measurement and asked respondents to directly assess work stability, by providing judgment on a seven-point scale from 1 = extremely unstable to 7 = extremely stable.

#### 3.4.2. Test of Reliability and Validity

The reliability, convergent validity, and discriminant validity of the scales were examined using Amos21.0 (IBM, Armonk, NY, USA) and SPSS 20.0 (IBM, Armonk, NY, USA). The internal consistency of the construct was evaluated. All Cronbach α values of the related scales and the minimum value in composite reliability (CR) were above 0.70, which is regarded as the threshold value [48]. Therefore, the reliability of our study was verified. The average variance extracted (AVE) values of related variables were above 0.5, which means convergent validity was good. The fit indexes of the model indicated that the overall model fit the data well ($\chi^{2}$ = 998.17; *p* < 0.001; GFI (goodness-of-fit index) = 0.956; CFI (comparative fit index) = 0.956; TLI (Tucker-Lewis index) = 0.926; IFI (incremental fit index) = 0.933; RMSEA (root-mean-square error of approximation) = 0.049). Taken together, these results provide satisfactory evidence of the reliability and convergent validity of our scales.

#### 3.4.3. Test of Common Method Bias and Multicollinearity

The common method bias (CMB) was not serious in this study. Firstly, AMEC was measured by the average of five health professionals from the same department, reducing the possibility of CMB. Secondly, as the other variables in this study were measured by the same respondents at one point in time, we conducted Harman’s single-factor analysis to test CMB. The result of exploratory factor analysis (EFA) suggested that all factors account for 61.2% of the total variance, but the first factor only represented 17.48%. Thirdly, the method of controlling the unmeasured single latent variable was used to test CMB. That is we treated the CMB as a latent variable added into the model and then loaded the latent variable on all items. By comparing the difference between the latent variable model and the original model, the obvious changes were not found. The variance inflation factors (VIFs) of the independent variables were all less than 2; thus, there was no evidence of the existence of CMB or multicollinearity problems.

## 4. Results

### 4.1. Descriptive Data Analysis

Table 1 presents descriptive information of the participants of the sample. More than 60% of health professionals in this sample are male. The age distribution is uniform, although the proportion of people aged from 30 to 34 is slightly higher. The vast majority of the participants graduated from junior college, and even 27.1% of them had a master’s degree or above. Besides, 76.3% of them have five years or more working experience, and 70.6% of them have professional titles.

Table 2 lists the means, standard deviations, and Pearson correlation coefficients between the main variables in this study. We preliminarily concluded that a significant positive correlation exists between AMEC and peer report intention (*r* = 0.327, *p* < 0.05). The impacts of attribution tendency and rewards on peer report intention were not verified and required in-depth regression analysis.

### 4.2. Hypothesis Testing

Quantitative data were analyzed using SPSS 20.0 with ordinary least squares (OLS) regression. Multiple stepwise regressions were performed to test the hypothesized relationships among variables, and Table 3 provides the regression results. All the incremental *R*^2^ values from one model to the next were statistically significant.

Hypothesis 1 predicted that positive AMEC would enhance peer report intention, while a negative AMEC would weaken it. As stated in Model 2 of Table 3, AMEC had a positive (*r* = 0.335,) and significant (*p* < 0.001) effect on peer report intention. The higher the value, the more positive the workplace AMEC; thus, H1 was supported.

As predicted by H2, attribution tendency mediated the link between AMEC and peer report intention. As proposed by Baron and Kenny, we conducted a four-step test to confirm the mediating effect of attribution tendency [49]. Firstly, AMEC alone showed a significant and positive impact on peer report intention (see Model2, Table 3, and discussion above). Second, as shown in Model 7, AMEC affected the mediator, attribution tendency, positively (*r* = 0.431) and significantly (*p* < 0.001). Third, attribution tendency had a significant positive effect on peer report intention (in Model 3, *r* = 0.325, *p* < 0.001). Fourth, when attribution tendency was controlled, the positive effect of AMEC on peer report intention was weakened. The regression coefficient of AMEC in Model 4 (0.166) was less than in Model2 (0.335), while the level of statistical significance decreased from *p* < 0.001 to *p* < 0.05. These results demonstrated that the effect of AMEC on peer report intention was partially mediated by attribution tendency. Therefore, H2 was supported.

Hypothesis 3 predicted that both contractual and extra-contractual rewards would promote peer report intention of on-looking health professionals. In Models 1 to 4, contractual reward showed positive and significant relationships with peer report intention (regression coefficient were all positive and *p*-values were all less than 0.05), while there was no evidence to confirm a positive effect of extra-contractual reward on peer report intention (all the regression coefficients were positive, but were not significant). Hence, only the contractual reward was effective in promoting peer report intention.

**Figure 3:**
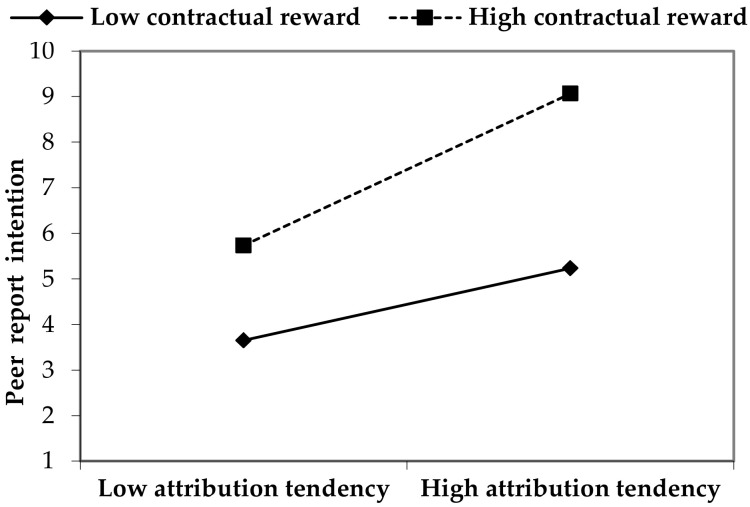
Moderating Effect of Contractual Reward on the Relationship between Attribution Tendency and Peer Report Intention.

For testing the interactive effects between rewards and attribution tendency, product terms were added into Model 5. As predicted by H4, attribution tendency and contract reward would have a positive and significant interaction effect (*r* = 0.233, *p* < 0.001; Figure 3). That is, if contractual reward and internal attribution tendency have effects simultaneously, then witnesses would have the strongest peer report behavior. Although attribution tendency and extra-contractual reward also had a significant interaction (*p* < 0.05), the regression coefficient of the product term was negative (−0.077) which was opposite to H4. Taken together, only the interaction effect between contract reward and attribution tendency was supported in this empirical study.

### 4.3. Robustness Checks

To further support our findings, we performed tests as follows: (1) The measure of a key variable (Peer Report Intention) was altered. The percentage of peer report possibility, which was represented by “you have a % chance of reporting AMEs during work”, was used to measure peer report intention in the hospital. (2) We reduced the sample. We mainly studied the mechanism through which AMEC in hospitals affects peer report intention. The attitudes of health professionals toward AMEs and peer report intention change with experience. Therefore, we analyzed the willingness of health professionals who had worked for one year or more to report AMEs by removing samples from individuals that had worked as a health professional for less than one year. (3) We conducted multiple stepwise regressions for different provinces. The result of this study is likely inaccurate as respondents were from different provinces. Therefore, the peer report intention of health professionals was analyzed separately by the province to exclude regional variation. The results of all the robustness checks showed that the regression coefficient and significance did not change significantly compared to the above, so the study results were found to be robust. Due to space limitations, these tables are not listed here but are available from us upon request.

## 5. Discussion

This study is a first step in exploring how and why hospital climate influences AME peer reports about AMEs. Although some specific cultural factors are thought to affect peer report intention and behavior [32], few studies have analyzed and tested the effects and action mechanisms therein. Considering the importance of climate for internal communication, as described in prior research [13], we developed a causal model that contains the mediating effect of attribution tendency and the moderating effects of rewards.

### 5.1. Hospital Climate and Peer Report Intention

We developed an effective measurement method of organizational atmosphere related to AMEs in China using the average of the peer reporting intent of five health professionals to reflect the AMEC of the hospital. The aim is to enhance the peer report intention of all AMEs, so the severity or form of these events was not emphasized. The model reflects the organizational context related to AMEs through the psychological climate of individuals within the organization. Although there are abundant and effective instruments for language and culture on AMEs [46,47], persons were unwilling to talk about peer reports on them. Therefore, we are the first to quantify the organizational context of AME reporting intent in hospitals and we verified that positive AMEC can enhance peer reporting intention.

Our findings highlight the necessity of improving the hospital climate from a practical perspective. Reporting AMEs is an essential component of hospital service improvement and it is influenced by organizational climate [24,50]. However, when faced with AMEs, witnesses have various options, including directly reporting to supervisors, righting wrongdoings or mistakes, implementing immediate remedies, and staying silent or ignoring the AE [9]. A positive climate means to more attention paid to AMEs and that precautionary measures and response mechanisms to AMEs have been implemented [23,51]. Furthermore, hospitals and their managers should expand solutions to the problem of “concealing the truth”, not limited to reacting by punishment. Particularly, supervisors’ attitudes and behaviors about AMEs may influence their access to critical information from subordinates.

### 5.2. Attribution Tendency

The results showed that attribution tendency mediates the link between AMEC and peer report intention. Although the hospital climate seems like one of the critical factors influencing peer report intention [9,23,32], few studies have empirically tested its influence or revealed the underlying action mechanisms, which is what we attempted to illustrate from the perspective of attribution. We propose that causal attribution on AMEs in a positive climate tends to be internal, which means some individuals should pay the price for the negative consequences of AMEs. In this context, report behavior is thought to benefit for problem-solving, because misconduct or defaults of individuals can be quickly remedied or corrected. The internal attribution tendency will enhance the reporting willingness of witnesses, and the test results of the mediating effect in Table 3 show that positive AMEC helps create this desired attribution. Therefore, a positive hospital climate encourages open discussion and communication about AEs, and then peer report intention increases as well. Taken together, hospitals and their managers should focus on causal attribution.

### 5.3. Contractual Reward

A high expected return will enhance the positive attitude toward these behaviors and will motivate these behaviors [1]. Few empirical studies have tested the validity of rewards’ effects on peer reports, though hospitals have established long-term systems of rewards [34]. Our findings confirm that contractual rewards effectively encourage peer reporting, especially for health professionals with internal attribution tendencies, although the effect of extra-contractual rewards has not been proven. Moreover, contractual reward also has an interactive effect with attribution tendency. Health professionals with internal attribution tendencies are likely to think someone should be penalized for AMEs, and people should not feel guilty or ashamed for uncovering and reporting AMEs. Immediate reports and responses help prevent AMEs from happening again and they mitigate the suffering of patients, especially when causes of AMEs are perceived to be located on the individual level. Therefore, rewards seem more attractive for those with internal attribution tendencies. The presence of rewards can lower the behavior threshold and strengthen the influence of attribution tendency. In practical terms, these results confirm the effectiveness of cash rewards and other benefits when informants confront potential risks and troubles resulting from peer reports [35,52]. However, the effect of extra-contractual reward and the interactive effect between extra-contractual reward and attribution tendency was not confirmed in this study. It was only inferred from survey data, and the unexpected results are worthy of future research.

There are other practical implications of the findings on the effects of rewards. Punishment is the most popular measure used in response to concealing AMEs in hospitals, but they may be aggravate the feeling of distress, frustration, and fear of health professionals [53,54]. Besides, it is difficult to confirm whether the witness knows the truth indeed and withholds the information deliberately. Rewards are another common and important incentive that should not be neglected [41,55]. Our empirical study confirms that a positive organizational climate improves peer report intention and reduces the possibility of adverse events through internal attribution tendency, and contract reward accelerates this process as an exogenous variable. Reward, as an incentive tool, helps to alleviate the fear of AMEs among health professionals by conveying the message that hospital holds a positive attitude towards AMEs, which reflects a positive organizational climate in hospitals. That is to say, we suggest that when hospitals make effort to solve the problems of concealing the occurrence of AMEs, they should not only create a positive atmosphere but also try to develop a formal reward plan to encourage employees to report. In addition to the theoretical and practical contribution, the findings also provide implications for future study. For example, it is necessary to probe into the mechanisms of action of rewards to grasp why these two kinds of rewards have different incentive functions.

### 5.4. Limitation

There are several potential limitations to this study. First, data and information from respondents may be masked, since the topics of peer reports and AEs are sensitive and subject to controversy in both theoretical research and practical situations, especially in China where social conformity and harmony have been advocated for thousands of years [40]. Although the questionnaires were anonymous, and most studies on peer reports or whistle-blowing have applied a similar method, respondents may still conceal their true feelings and ideas, which may further lead to potential bias in the results. Future studies should focus on how to design proper methodologies and procedures to capture the feelings of the respondents.

AEs can be divided into several types, and some scholars have applied other scenarios in their studies, such as employee theft, other unethical behaviors, and industrial or safety accidents [1,13]. We grouped all of AMEs into the same category, which led to another potential limitation of this study: an ambiguous definition of AMEs. Some scholars have argued that the severity and other characteristics of AEs significantly influence peer report intention [9]. The robust findings and conclusions in this study should be tested using more subcategories and specific scenarios. On the one hand, health professionals in this study came from hospitals in the Yangtze River Delta region of China, and other scholars have explored the possibility of teamwork in reducing the error rate of emergency care who used European data [56]. Therefore, future research can be to study the role of organizational climate in reducing AMEs in other regions and countries which can enhance the reliability of the results. On the other hand, various AMEs differ in severity [6], and concealing serious medical malpractice will cause enormous loss to patients and hospitals, and could even result in the imprisonment of responsible persons to prison. Compared with the slight medical error, timely peer report of serious AMEs seems more valuable to alleviate patient suffering but informants are more likely to experience revenge. The severity of AMEs must be considered in peer report intention studies.

## 6. Conclusions

In recent years, researchers have primarily focused on how to reduce the rate of AMEs, and have begun to study the effect of organizational climate on the peer report of AME. The present study collected questionnaires from health professionals of various hospitals in the Yangtze River Delta region in China and conducted an empirical analysis. This deepens the understanding of how positive and negative AMEC affect peer reports intention. The number of AMEs is also associated with attribution tendency and contract reward, and the incidence of adverse events in the hospital can be reduced by building a positive AMEC and setting up effective contract reward.

## Figures and Tables

**Table 1:** Sample description.

Health Professionals	Number (*N* = 503)	Proportion	Health Professionals	Number (*N* = 503)	Proportion
**Gender**			**Years of working**		
Man	304	60.4%	Under 4	119	23.7%
woman	199	39.6%	5–9	153	30.4%
**Age**			10–19	121	24.1%
Under 30	121	24.0%	20–40	110	21.8%
30–34	146	29.0%	**Professional title**		
35–39	125	24.9%	No title	148	29.4%
Over 40	111	22.1%	Junior	161	32.0%
**Education**			Middle	132	26.3%
Below junior college	36	7.1%	Senior	62	12.3%
Junior college	134	26.6%			
Undergraduate	197	39.2%			
Master and above	136	27.1%			

**Table 2:** Means, Standard Deviations and Correlations.

Variable	Mean	S.D.	1	2	3	4	5	6	7	8	9	10
1 Peer report intention	3.4592	1.67767	1									
2 Education	2.7117	0.73772	−0.009	1								
3 Seniority	5.8827	5.69687	0.186 **	0.001	1							
4 Ownership	1.3360	0.47280	−0.072	−0.042	0.069	1						
5 Scale	2.8211	0.54644	−0.023	0.139 **	0.080	0.025	1					
6 Work Stability	3.3917	1.69828	0.295 **	−0.019	−0.124 **	0.037	−0.070	1				
7 Contractual Reward	3.0676	1.48143	0.191 **	0.054	−0.033	−0.021	−0.128 **	0.027	1			
8 Extra-contractual Reward	3.0378	1.54757	0.171 **	0.126 **	0.018	−0.036	−0.192 **	0.072	0.638 **	1		
9 AMEC	3.5968	1.23090	0.327 **	0.003	0.137 **	−0.074	0.073	0.083	0.152 **	0.121 **	1	
10 Attribution tendency	3.8210	1.27149	−0.329 **	−0.063	−0.206 **	0.099 *	0.063	0.027	−0.012	−0.064	−0.312 **	1

*n* = 503. Two-tailed tests; * *p* < 0.05; ** *p* < 0.01.

**Table 3:** Regression Results for Peer report intention and Attribution Tendency.

Variable	Peer Report Intention					Attribution Tendency	
	Model 1	Model 2	Model 3	Model 4	Model 5	Model 6	Model 7
Intercept	1.736 *** (0.520)	0.904 ^†^ (0.520)	3.123 *** (0.527)	2.298 *** (0.554)	1.892 * (0.697)	3.533 *** (0.421)	4.373 *** (0.436)
Education	−0.052 (0.095)	−0.038 (0.091)	−0.096 (0.090)	−0.078 (0.089)	−0.059 (0.087)	−0.111 (0.077)	−0.128 ^†^ (0.074)
Seniority	0.069 *** (0.012)	0.058 *** (0.012)	0.050 *** (0.012)	0.046 *** (0.012)	0.049 *** (0.011)	−0.049 *** (0.010)	−0.044 *** (0.010)
Ownership	−0.341 * (0.145)	−0.265 ^†^ (0.140)	−0.227 (0.138)	−0.193 (0.136)	−0.212 (0.140)	0.291 * (0.117)	0.223 * (0.112)
Scale	0.046 (0.130)	−0.030 (0.126)	0.120 (0.123)	0.054 (0.122)	0.043 (0.123)	0.187 ^†^ (0.105)	0.257 * (0.101)
Work Stability	0.317 *** (0.041)	0.291 *** (0.039)	0.318 *** (0.039)	0.300 *** (0.038)	0.310 *** (0.039)	0.003 (0.033)	0.019 (0.030)
Contractual Reward	0.187 ** (0.060)	0.148 * (0.058)	0.197 *** (0.057)	0.167 ** (0.056)	0.109 ** (0.037)	0.025 (0.048)	0.054 (0.049)
Extra-contractual Reward	0.045 (0.059)	0.034 (0.057)	0.028 (0.056)	0.023 (0.055)	0.065 (0.098)	−0.042 (0.048)	−0.035 (0.049)
AMEC		0.335 *** (0.055)		0.166 * (0.076)	0.252 *** (0.053)		0.431 *** (0.052)
Attribution tendency			0.325 *** (0.054)	0.393 *** (0.053)	0.193 (0.121)		
Attribution × contract					0.233 *** (0.049)		
Attribution × extra-contract					−0.077 * (0.032)		
*R* ^2^	0.184	0.240	0.266	0.292	0.306	0.068	0.153
Adjusted *R*^2^	0.172	0.228	0.254	0.279	0.290	0.055	0.139

*n* = 503. Unstandardized coefficients shown with standard errors in parentheses. ^†^ *p* < 0.10; * *p* < 0.05; ** *p* < 0.01; *** *p* < 0.001.

## Data Availability

Data is contained within the article or Appendix A.

## Appendix A

**Table A1:** The details of the instrument.

Construct/Measurement Item	Reference
**Peer-reporting intention**	Refer to the measurement of Trevino and Victor [1] and Jacobs [24] to measure report willingness directly
I will report AMEs that happened in the hospital if I know.	
**AMEC**	Refer to the nine-item and seven-point scale of Dedobbeleer and BeLand [46] and modified in detail according to the study of Curran et al. [47].
(1) how much your supervisors seem to care about AMEs?	
(2) how much management commitment to AMEs in your hospital?	
(3) how important preventing AMEs to hospital management?	
(4) do you regard AMEs responding as an integrated part of the job?	
(5) whether do you perceive you would be involved in an AME in the next 12 months?	
(6) do you feel pleasant communicating with your supervisor about AMEs?	
(7) does your boss take the suggestions about reducing the incidence of AMEs?	
(8) do you think the hospital treats you and your colleagues fairly?	
(9) do you have that “we are family” feeling with your colleagues?	
**Attribution Tendency**	Refer to the judgments and suppositions of AMEs’ cause, and modified the scale according to Hofmann and Stetzer’s [13] study
(1) AMEs are usually caused by health professionals’ carelessness and malpractice (reverse-coded)	
(2) it’s hard for health professionals to influence and control whether AMEs exist or not	
(3) AMEs are usually caused by time pressure to get the job finished more quickly (reverse-coded)	
(4) AMEs are usually caused by poor choices on the part of the health professional(reverse-coded)	
(5) AMEs are usually caused by the situation in general (reverse-coded)	
(6) AMEs are usually caused by the skill of the health professional in general (reverse-coded)	
**Contract Reward**	Transferred from concept connotation
informants will get a cash reward for peer-report on AMEs.	
**Out-contract Reward**	
peer-report on AMEs will bring informants favorable impression of supervisors and can help for promotion in future.	
